# Integrating cervical cancer with HIV healthcare services: A systematic review

**DOI:** 10.1371/journal.pone.0181156

**Published:** 2017-07-21

**Authors:** Louise Sigfrid, Georgina Murphy, Victoria Haldane, Fiona Leh Hoon Chuah, Suan Ee Ong, Francisco Cervero-Liceras, Nicola Watt, Alconada Alvaro, Laura Otero-Garcia, Dina Balabanova, Sue Hogarth, Will Maimaris, Kent Buse, Martin Mckee, Peter Piot, Pablo Perel, Helena Legido-Quigley

**Affiliations:** 1 Centre for Tropical Medicine and Global Health, Nuffield Department of Medicine, University of Oxford, Oxford, United Kingdom; 2 Saw Swee Hock School of Public Health, National University of Singapore, Singapore, Singapore; 3 London School of Hygiene & Tropical Medicine, London, United Kingdom; 4 Universidad de Lleida, Lleida, Spain; 5 Nursing Section, Faculty of Medicine, Universidad Autónoma de Madrid, Madrid, Spain; 6 Ciber of Epidemiology and Public Health (CIBERESP-ISCIII) Madrid, Spain; 7 London Borough of Waltham Forest, London, United Kingdom; 8 Haringey Council. Civic Centre, London, United Kingdom; 9 Strategic Policy Directions, UNAIDS, Geneva, Switzerland; Universidade Estadual de Maringa, BRAZIL

## Abstract

**Background:**

Cervical cancer is a major public health problem. Even though readily preventable, it is the fourth leading cause of death in women globally. Women living with HIV are at increased risk of invasive cervical cancer, highlighting the need for access to screening and treatment for this population. Integration of services has been proposed as an effective way of improving access to cervical cancer screening especially in areas of high HIV prevalence as well as lower resourced settings. This paper presents the results of a systematic review of programs integrating cervical cancer and HIV services globally, including feasibility, acceptability, clinical outcomes and facilitators for service delivery.

**Methods:**

This is part of a larger systematic review on integration of services for HIV and non-communicable diseases. To be considered for inclusion studies had to report on programs to integrate cervical cancer and HIV services at the level of service delivery. We searched multiple databases including Global Health, Medline and Embase from inception until December 2015. Articles were screened independently by two reviewers for inclusion and data were extracted and assessed for risk of bias.

**Main results:**

11,057 records were identified initially. 7,616 articles were screened by title and abstract for inclusion. A total of 21 papers reporting interventions integrating cervical cancer care and HIV services met the criteria for inclusion. All but one study described integration of cervical cancer screening services into existing HIV services. Most programs also offered treatment of minor lesions, a ‘screen-and-treat’ approach, with some also offering treatment of larger lesions within the same visit. Three distinct models of integration were identified. One model described integration within the same clinic through training of existing staff. Another model described integration through co-location of services, with the third model describing programs of integration through complex coordination across the care pathway. The studies suggested that integration of cervical cancer services with HIV services using all models was feasible and acceptable to patients. However, several barriers were reported, including high loss to follow up for further treatment, limited human-resources, and logistical and chain management support. Using visual screening methods can facilitate screening and treatment of minor to larger lesions in a single ‘screen-and-treat’ visit. Complex integration in a single-visit was shown to reduce loss to follow up. The use of existing health infrastructure and funding together with comprehensive staff training and supervision, community engagement and digital technology were some of the many other facilitators for integration reported across models.

**Conclusions:**

This review shows that integration of cervical cancer screening and treatment with HIV services using different models of service delivery is feasible as well as acceptable to women living with HIV. However, the descriptive nature of most papers and lack of data on the effect on long-term outcomes for HIV or cervical cancer limits the inference on the effectiveness of the integrated programs. There is a need for strengthening of health systems across the care continuum and for high quality studies evaluating the effect of integration on HIV as well as on cervical cancer outcomes.

## Introduction

Cervical cancer is a public health priority in many parts of the world and remains among the leading causes of cancer in women. Most (85%) cases occur in low-income countries [1] and, in 2012, 90% of deaths were in low- and middle-income countries [2]. Most of these deaths could have been prevented through universal access to comprehensive cervical cancer prevention and control programs [2]. Indeed, screening and treatment of pre-cancerous lesions to prevent cervical cancer is one of the World Health Organization’s ‘best buys’ for the prevention and control of non-communicable diseases [3].

Women living with HIV have higher risk of invasive cervical cancer, reflecting both immunosuppression caused by HIV infection and shared risk factors [4]. They also have higher prevalence of persistent HPV infection, the primary cause of cervical cancer [5] compared with those HIV negative [6]. The natural history of HPV infection has a slow, 10–20 year progression to pre-cancer in immunocompetent women; however, women living with HIV progress more frequently and quickly to pre-cancer and cancer [2].

There are large inequities in access to effective cervical cancer screening and treatment, with corresponding differences in the risk of invasive disease [2], with screening coverage in low- and middle-income countries only 19% overall, but much lower in some (e.g. 1% in Bangladesh) [7].

Cervical cancer screening requires a reliable health infrastructure for implementation, sustainability and achievement of coverage of more than 70–80% to be effective [8]. Even though the number of new HIV infections is decreasing in most populations, it remains a major threat amongst vulnerable groups worldwide and particularly in parts of Africa; nearly half of new HIV infections occur among people living in Nigeria, South Africa and Uganda, where HIV and AIDS constitute the number one cause of life-years lost [9]. Consequently, many countries are now coping with the dual burden of HIV and cervical cancer [10].

Given the efficacy of antiretroviral treatment, the increased risk of cervical cancer among women living with HIV, and the low access to screening in several countries with high HIV prevalence, improved systems for screening and treatment are needed [11]. The World Health Organization’s guidelines for cervical cancer control recommends cervical cancer screening as soon as girls or women are tested positive for HIV, regardless of age, using visual inspection with acetic acid (VIA), HPV test or cytology depending on available resources, and cryotherapy and loop electrosurgical procedure (LEEP) for treatments [2, 12]. Moreover, inclusion of HIV counselling and testing (HCT) into cervical cancer screening programs, and vice versa, is recommended for all countries with high HIV prevalence, together with special efforts to reach vulnerable populations, such as women living with HIV [2]. Yet although integration is intuitively appealing, relatively little is known about the models of integration and factors that facilitate or hinder integration in different contexts.

Low-cost screening techniques, such as VIA and cryotherapy, have been proposed for women living with HIV in low-resource settings [13]. However, to our knowledge there is no systematic review of integration of cervical cancer screening with HIV care. To address this gap, we systematically reviewed the literature describing and evaluating interventions that sought to integrate cervical cancer screening and treatment with HIV, reporting outcomes where available, concluding with recommendations for future research and policies.

## Methods

### Definitions

We followed PRISMA guidelines [14] as part of a larger systematic review on integration of HIV and non-communicable diseases (See S1 Table for PRISMA checklist). Where possible we follow the PICOS structure for study characteristics, adapted for purely descriptive studies. Drawing on the definitions proposed by Briggs, Atun et al and Legido-Quigley et al [15–18], the concept of integration and its key attributes are described in Box 1 [19]. We also drew on a typology of integration whereby *service* integration involved different clinical services being integrated using teams or multidisciplinary professionals and *clinical* integration involved care being integrated into a single or coherent process within and/or across professions [20]. This review covers populations accessing health services and healthcare workers, and interventions integrating cervical cancer screening and/or treatment services with HIV testing and/or treatment services. Compared to non-integrated services for cervical cancer screening and/or treatment and HIV testing and/or treatment. Due to the descriptive nature of the review any outcomes reported by the original studies were included, including barriers and facilitators to the intervention.

**Box 1. Domains of integration (drawing on [19])**

○ Integration across disease programs (clinically related diseases)
○ Integration across disease programs (clinically different diseases), for example:
  ■ Integration across high burden conditions (e.g. HIV, malaria, TB) to reduce impact of co-infections
○ Integration between vertical (disease-specific) and horizontal (system-wide) programs, which may involve:
  ■ Integration of interventions within a ‘building block’ of the health system (e.g. integrated staff training, financial and organizational management etc.)
  ■ Integration across one or more building blocks of the health system (e.g. human resource policies and governance initiatives)
  ■ Integration across ‘service functions’: of inputs, of different levels of service delivery, of management and operational decisions and technology
○ Integration across public health programs and health service interventions, for example:
  ■ Integration between MNCH, family planning, through trained community health workers, and health promotion.
○ Integration across activities in the health systems and other sectors (e.g. treatment combined with educational interventions and community mobilization)

### Inclusion criteria

We included all quantitative and qualitative studies describing or evaluating a management or organizational change policy or intervention, implemented within an existing health system, aiming to integrate HIV and cervical cancer screening and/or treatment at service delivery level. Services could be provided in health facilities or in the community. We did not exclude reports based on study design; nor did we require them to include outcome measures. We imposed no language, publication date, or publication status restrictions. Conference abstracts were included as they are an important source of unpublished studies. No studies were excluded based on assessment of bias.

### Search strategy

The search strategy and terms were developed collaboratively with an information specialist, and were consistent with methods adopted by other authors who have conducted systematic reviews on health services integration [15, 16]. The following electronic databases were searched from inception until February 2014: Global Health, Medline and Embase. Key words (MeSH terms) and free text terms were developed for 3 themes: HIV, integration and chronic diseases and then combined in the search strategy, after which the papers on integration of HIV and cervical cancer were identified. The search terms used for Medline are shown in Box 2. In addition, we searched the following databases using a simplified search strategy to ensure maximum yield of papers from low and middle income countries: Cochrane library, LILACs, Africa Wide, WHOLIS and abstracts from the International AIDS Society (IAS) Online Resource Library from 2006 to 2015, the HIV Implementers meetings from 2007 to 2012 and International conferences on non-communicable diseases. We conducted an updated search until December 2015 using Global Health, Medline and Embase and references of included papers were searched manually.

**Box 2. Search Strategy used for Medline, Embase and Global Health via Ovid (adapted to only include cervical cancer terms)**

Database: Embase <1980 to December 2015>, Global Health <1910 to December 2015>, Ovid MEDLINE(R) <1946 to December 2015>

1. ((vertical or horizontal or integrat* or coordinat* or co-ordinat* or link*) and (program* or care or service*)).mp. or delivery of health care, integrated/ or primary healthcare/
2. exp HIV infections/ or HIV.mp. or Human immunodeficiency virus.mp. or "HIV/aids".mp.
3. (All introduced in a separate line) chronic disease/ or long-term care/ or ((chronic* or persistent or long* term or ongoing or degenerative) adj3 (disease* or disab* or ill* or condition* or health condition* or medical condition*)).tw. or long* term care.tw. or (non-communicable disease* or NCD).tw. or exp neoplasms or (cancer* or oncolog* or neoplasm* or carcinom* or tumo?r* or malignan* or cervical cancer).tw.
4. 1 and 2 and 3

### Search and retrieval of studies

Two reviewers independently reviewed the list of articles retrieved by the electronic database search, based on title or title and abstract, to identify those meeting the inclusion criteria. If either of the two reviewers considered a study potentially eligible, the full text of this article was retrieved for further assessment. There were no studies identified in languages other than English. The retrieved full texts were assessed independently for inclusion by two reviewers. Disagreements were resolved in discussion with a third reviewer.

### Data synthesis

Two reviewers independently extracted data from included studies using standardized forms. Differences in data extraction or interpretation of studies were resolved by discussion and consensus. We extracted data from the results and discussion sections of both quantitative and qualitative studies including information on: (1) study characteristics including study design, setting and sample size, (2) participants characteristics including age, gender, ethnicity and country of origin, (3) integration activities of the program or intervention, (4) results and type of outcome measure including clinical, procedural and behavioral outcomes, and (5) the facilitators and barriers to integration activities as discussed in each study. The data were compared across studies and any conflicting findings noted and, where possible, explained. We conducted a narrative synthesis of the findings. Studies covering the three different models are summarized and presented in the following sections and illustrated in more details using examples from some of the more complex studies for each model.

### Risk of bias assessment

Studies which presented evaluative rather than purely descriptive data were independently assessed by two reviewers for risk of bias using the Cochrane risk of bias tool for randomized studies[21] and a simple pro-forma for observational studies with three domains: selection bias, information bias (differential misclassification and non-differential misclassification) and confounding. Each domain was assessed as low, unclear or high. Studies were considered evaluative only if there was an alternative group with which to compare the integration intervention. We classified studies that had a low risk of bias in all domains as having a low overall risk of bias. Studies that had a high or unclear risk of bias in one or more domains were classified as having an overall high or unclear risk of bias.

## Results

11,057 records were identified through database searching. 7,616 articles, remaining after exclusion of duplicates, were screened by title and abstract for inclusion. 340 papers involved one or more NCDs. After screening of the full papers 155 articles were included in total (S1 Fig). 21 of these articles, representing 23 studies, covered integration of HIV and cervical cancer and were included in this review (S1 Fig). All were in English. Five of the studies were conference presentations [22–26]. Due to the heterogeneity in study designs, intervention types, participants, and outcomes, we did not conduct a meta-analysis but instead present a summary of the articles, and a synthesis of their results and outcomes where available.

### Characteristics of included studies

Of the 23 included studies, 15 were cross-sectional, four were cohort studies, three were retrospective record reviews, and one was a before and after study. 17 studies were set in Africa, four in South America, one in Asia and one in Europe (S2 Fig). Most were conducted in lower-middle-income countries (n = 9), followed by low-income countries (n = 7), and upper-middle-income (n = 6), with only one study from a World Bank classified higher-income country [27]. 2 displays a geographical representation of the models reported. Almost all articles described the introduction of new services that a health facility had not previously offered; only one study assessed the integration of existing services. [28] All but one study described integration of cervical cancer screening, with or without treatment, into existing HIV services. One described integration of HIV screening into an existing cervical cancer screening service. [29] Several studies described the cervical cancer services offered in detail, but none described the HIV care offered or outcomes besides HIV screening. Eight programs included only women living with HIV [4, 24, 28, 30–33] only, the others including all women accessing the clinics.

### Models of integration

Three models of integrated care were identified, based on descriptions of services provided. The first was within-clinic integration using internal staff. In this model, the existing clinic structure and staff was used to incorporate a new set of services to complement services that are already provided. In a second model, integration was achieved through co-location. HIV and cervical cancer services were provided to the patient through coordination of care between different specialists or clinics within the same health care facility. The third model involved complex programs of integration and coordination, including programs that integrated services by involving a range of different types of health workers (often from community health workers to clinical specialists) and facilities, and established systems to ensure clinical coordination and follow-up of patients (S3 Fig). In all three models, we found different intensities of services being provided, from cervical cancer screening only to screening and treatment of minor to larger lesions (Table 1).

**Table 1:** Type of integration and cervical cancer services provided. The table shows the integration models described in the included studies.

Integration model	CaCx Services	CaCx methods	Setting	Author and Country
**Within clinic integration using internal staff**	**CaCx screening**	VIA	HIV clinics	• Morgan 2014 *[Guyana]*
		PAP	HIV / ID clinics	• Sirivongrangson 2007* *[Thailand]*
			GUM clinic	• Ibrahim 2013 *[England]*
	**CaCx ‘screen and treat’ minor lesions**	VIA + Cryotherapy	HIV clinics	• Ekong 2013 *[Uganda]*
			Family planning clinics	• Moon 2012 *[Mozambique]*
			Mobile HIV clinics	• Mulenga 2012 *[Zambia]*
	**CaCx ‘screen and treat’ larger lesions**	VIA + Cryotherapy + LEEP	HIV clinics and RCH clinics	• Anderson 2015 *[Côte d’Ivoire*, *Guyana*, *and Tanzania]* • Martin 2014 *[Guyana]*^
			HIV clinics	• Huchko 2011 *[Kenya]*
**Coordination between co-located clinics/specialists**	**CaCx screening**	VIA	HIV clinic and RCH clinic	• Odafe 2013 *[Nigeria]*
			ART and blood giving clinics	• Horo 2012 [Côte d’Ivoire]**
		PAP+ colposcopy	HIV/ID clinic	• Fink 2012 *[Argentina]*
	**CaCx ‘screen and treat’ minor lesions**	VIA + Cryotherapy	Cervical Cancer Prevention Program clinics in HIV clinics	• Mwanahamuntu 2013 [Zambia]*** • Ramogola-Masire 2012 *[Botswana]*
			Public health clinics	• Parham 2010 [Zambia]***
	**CaCx ‘screen and treat’ larger lesions**			
**Complex program of integration and coordination**	**CaCx screening**	PAP	HIV / ID clinics	• McCree-Hale 2011 *[Tanzania]*
	**CaCx ‘screen and treat’ minor lesions**	VIA + Cryotherapy	Family planning, and child and maternal health clinics	• Khozaim 2014 *(Kenya)* • Plotkin 2014 *[Tanzania]*
	**CaCx ‘screen and treat’ larger lesions**	VIA + Cryotherapy + LEEP	HIV clinics and/or Public health clinics	• Mungo 2013 *[Kenya]* • Pfaendler [Zambia] • Shiferaw,2016 *[Ethiopia]*^^

^LEEP available in two of the 18 sites

^^LEEP available at some of the sites or by referral

*PAP and HPV testing

**VIA and VILI

***VIA and digital camera

CaCx: cervical cancer, VIA: visual inspection with acetic acid, PAP: Papanicolaou test, ID: infectious diseases, GUM: genitourinary medicine, LEEP: loop electrosurgical excision procedure, RCH: reproductive and child health.

### Model 1: Within-clinic integration using existing staff

This model was described in nine papers covering 11 studies describing integration of cervical cancer screening and different levels of treatment, into existing clinics providing HIV services. The integration and services offered are described in Table 2. Most programs (n = 10) were set in low-resource settings. Only two of the studies formally evaluated integration [30, 34]. Four studies briefly described additional integration of gynecology, sexually transmitted diseases (STDs) and/or family planning [5, 28, 30, 35]. In nine studies, the main clinical outcomes were reported as numbers screened for cervical cancer. Four reported proportions screened (ranging from 67–87%) and nine reported cervical cancer screening results (Table 3). Three studies reported high loss to follow up for referrals or annual follow-up appointments [5, 26, 28]. None of the studies described the HIV services offered and none presented clinical HIV outcomes. Three studies reported uptake of STD screening [28, 33, 35].

**Table 2:** Model 1—Within clinic integration using internal staff.

Study	Study design	Setting	Model of care	Integration	Screening n (% of those offered)	HIV positive	Treatment coverage	Selection bias
Morgan 2014	Cross-sectional	The National Care and Treatment referral centre (NCTC), *Guyana*	Single-site approach: VIA screening	VIA provided as a baseline test for all HIV positive women and reinforced by NCTC health team, which also promotes annual VIA screening. Also extends to all women.	1,831	49%	*NA*: *Screening only*	N/A
Sirivongrangson 2007	Cross-sectional	An urban infectious disease clinic and an STI clinic, *Thailand*	Screening: Pap test, HPV DNA test, and screening and treatment of STIs. Referral of those with abnormal cervical cytology.	HIV-infected women attending either an infectious disease clinic or a STI clinic were screened.	150 (70.8%) at infectious disease clinic and 60 (100%) at the STI clinic.	100%	*NA*: *Screening only*	N/A
Ibrahim 2013	Retrospective record review	A hospital genitourinary medicine department, *the UK*	Screening using smear test and cytology. Referred for colposcopy according to local and national guidelines.	CaCx screening integrated into a genitourinary medicine clinic for HIV positive women.	101 (78%)	100%	Following the initial smear, all women were appropriately followed up with annual cytology or referred for colposcopy according to local and national guidelines. 22 patients were lost to follow-up after initial cytology.	N/A
Ekong 2013	Retrospective record review	Five rural ART clinics, *Uganda*	‘See and Treat’: VIA and cryotherapy, advanced cases referred.	Existing healthcare workers trained to provide CaCx services.	1,088	19%	53.6% (15/28) HIV-positive and VIA-positive women were treated with cryotherapy; 46.4% were referred.	N/A
Moon 2012	Cross-sectional	Four rural family planning health facilities and one referral hospital, *Mozambique*	‘See and Treat’: VIA and cryotherapy, advanced cases referred. LEEP and surgery were provided at the provincial hospital for serious cases.	CaCx screening, family planning, HIV VCT, and STI and gynecological screening all performed during one visit in the same physical space. Technical assistance infrastructure of a HIV program used to introduce CaCx services.	4,651	12.5%	61% (221/380) of cryotherapy eligible women received same day treatment–increasing from 53% during the first quarter to 96% during the last quarter.	N/A
Mulenga 2012	Cross-sectional	14 Zambia Defense Force mobile HIV VCT service units, *Zambia*	‘See and Treat’: VIA and cryotherapy, advanced cases referred.	Screening provided on an opt-out bases to women accessing mobile HIV VCT services.	560 (67%)	20%	11% (62/560) were eligible for onsite cryotherapy and were treated immediately, while 5% (26/560) were referred, of these 92% (24/26) completed the referral.	N/A
Anderson 2015	Cross-sectional	24 HIV clinics and 23 reproductive and child health clinics in national, regional, and district hospitals, and health centers, *Côte d’Ivoire*, *Guyana*, *and Tanzania*	‘See and Treat’: VIA and cryotherapy, ineligible referred for LEEP, advanced cases referred.	Existing healthcare workers trained to provide CaCx services. Shared training protocols and multiple types of staff involved.	34,921	26%	85% (2,508) of eligible women received cryotherapy during the same visit; only 234 (52%) of those who postponed returned for treatment; 622 (17%) VIA-positive women were referred for excisional treatment.	N/A
Martin 2014	Retrospective record review	18 CaCx prevention sites, including 10 HIV care and treatment sites, *Guyana*	‘See and Treat’: VIA and cryotherapy, ineligible referred for LEEP, advanced cases referred, referred patients followed up. Counselling and education.	Physicians and non-physicians trained to provide CaCx screening services. Development of treatment guidelines with Ministry of Health and stakeholders.	21,597	8%	85% (1938) of women eligible for cryotherapy received immediate treatment. Of the 347 women who postponed cryotherapy, 62% returned for treatment, while 38% were lost to follow-up. Half (1,027) of VIA+ women treated with cryotherapy LEEP returned for a 1-year follow-up screening.	Unclear risk of bias
Huchko 2011	Retrospective record review	District hospital and HIV clinics, *Kenya*	VIA and colposcopy, LEEP treatment, advanced cases referred. Outreach, awareness, and education campaign.	CaCx screening offered as part of routine care at HIV clinics. Full clinic involvement and training. Coordination with local experts and external pathologists.	3,642 (87%)	100%	531 (15%) underwent colposcopy for either positive or unsatisfactory VIA; 243 LEEPs were performed. Eight women (0.1%) were referred for radiation therapy or surgery.	Unclear risk of bias

Abbreviations: VIA: visual inspection with acetic acid, NA: not applicable, STI: sexually transmitted infection, HPV: human papilloma virus, CaCx: cervical cancer, LEEP: loop electrosurgical excision procedure, VCT: voluntary counselling and testing.

**Table 3:** Types of outcomes reported.

Type of model	Patient Outcomes	N	Process Outcomes	N
Within clinic integration	Numbers offered CaCx screening	9	Proportion screened within 1 year of HIV diagnosis	1
	Proportion accepting CaCx screening	4	Proportion followed up annually	2
	CaCx screening results	9	Number of staff trained	3
	Proportion offered cryotherapy	6	Loss to follow up	5
	Proportion referred for larger lesions and treatment	6	Screening uptake by type of clinic or region	3
	Proportion offered colposcopy	3	Proportion treated with cryotherapy same day	3
	Proportion taking up colposcopy	1	Complications/severe adverse events	2
	Pathology results	3	VIA positive rates over time	1
	Cancer diagnosis	4	Proportion of service providers offering screening over time by type of provider	1
	Reasons for declining CaCx screening	1	Proportion screened for CaCx versus national screening program over time	1
	CD4 counts	2	Staff satisfaction	1
	Proportion on HAART/ART	1	Provider barriers	4
	Proportion with STI	3		
	Perceived patient barriers	2		
	Proportion with high risk HPV infections/types of HPV	1		
Coordination through colocation	Numbers offered CaCx screening	5	Loss to follow up	2
	Proportion accepting CaCx screening	1	Proportion undergoing cryotherapy same day	1
	CaCx screening results	5	Proportion returned for follow up	1
	Proportion on HAART/ART	2	Probability model of program effectiveness	1
	Proportion referred for further CaCx diagnostics or treatment	4	Sensitivity and specificity of nurse screening assessment	1
	Patient barriers for uptake of support	1		
	CaCx pathology results	3		
	Cancer diagnosis	2		
	Proportion CaCx screen positive at follow up screening	1		
Complex coordination	Numbers offered CaCx screening	6	Loss to follow up	3
	Proportion accepting CaCx screening	3	Proportion diagnosed using Colposcopy vs. LEEP	1
	CaCx screening results	5	Probability model of number of cancer cases prevented	1
	Proportion taking up CaCx treatment	1	Numbers screened for HIV over time	1
	Proportion referred for CaCx diagnostics and treatment	3	Proportion followed up with repeat CaCx screening over time and outcomes	1
	Proportion referred for larger CaCx lesions and treatment	1	Hazard of recurrence of CaCx	1
	CaCx pathology results	1	Proportion followed up annually	1
	Cancer diagnosis	3	Proportions followed up	1
	Numbers offered HIV screening	1	Proportion accepting CaCx screening by type of clinic or region	1
	Proportion accepting HIV screening	1	Proportion treated with cryotherapy same day	1
	Reasons for not offering HIV screening	1	Numbers screened for CaCx over time	1
	Reasons for declining HIV screening	1		
	Complications	1		
	Patient barriers to uptake	3		

Abbreviations: ART: Antiretroviral Therapy, CaCx: cervical cancer, HAART: Highly Active Antiretroviral Therapy, LEEP: Loop Electrosurgical Excision Procedure, STI: sexually transmitted infections

N: The number of studies that reported this outcome, by model of integration

A cross-sectional study from Mozambique [35] described integration of cervical cancer services with PEPFAR-funded rural clinics and a hospital, with training of existing child health nurses in VIA and cryotherapy. Women were offered cervical cancer screening together with screening for STDs and gynecology pathologies in a single visit. Prior to integration, there was no access to cervical cancer screening. The service screened twice as many women in the first year (n = 4,651, 13% HIV+) for cervical cancer than envisaged in the Ministry of Health target. The VIA+ rate was 8% (n = 380). 61% of VIA+ women underwent cryotherapy the same day and 4% required referral. By the end of the year, 96% were receiving treatment the same day, without any adverse events. 5% (n = 218) of women were diagnosed clinically with STIs; 98% (n = 214) received treatment.

Another cross-sectional study evaluated the integration of ‘screen-and-treat’ cervical cancer services in 24 HIV and 23 reproductive and child health clinics (RCHCs) in Cote d’ Ivoire, Guyana and Tanzania with training of existing staff in cervical cancer screening and treatment. The integrated services screened >34,000 women for cervical cancer (2009–2012). [5] 10% (n = 3,580) of women screened were VIA+ and 85% (n = 2,508) of eligible women received cryotherapy during the same visit. Immediate treatment of small-to-large lesions in a single-visit reduced loss to follow up. In contrast, only 52% (n = 234) of women who postponed treatment returned. In multivariate analysis, controlling for a range of factors, women living with HIV had higher odds of being VIA+ (OR 1.95, 95% CI 1.76, 2.16, P<0.0001) and of having large lesions requiring referral (OR 1.93, 95% CI 1.49, 2.51, P< 0.0001) compared to HIV- women. The risk of complications was <1%.

A cross-sectional study set in Zambia[26] evaluated uptake of a new cervical cancer screening and treatment integrated into mobile outreach vans delivering HIV screening and support. The authors reported a 67% uptake (n = 560, 20% were HIV+); 16% (n = 88) had abnormal cervical lesions. 11% (n = 62) screened and eligible for cryotherapy underwent immediate treatment. 5% (n = 26) were referred for treatment of larger lesions of which 92% (n = 24) completed the referral.

### Model 2: Coordination through co-location clinics/specialties

Six papers described this model of integration (Table 4); five programs were set in Africa [4, 31, 36–38], and one in South America [23]. Five of the studies described integration through co-location of cervical cancer and HIV clinics; one program used a model based on mobile gynecologists who provided weekly cervical cancer services in HIV clinics. Most studies presented data on numbers of women screened for cervical cancer, one reported the proportion accepting screening, and five studies reported screening results. Two studies reported cancer diagnoses. Two studies reported high loss to follow up,[36, 38] with one reporting higher rates amongst women living with HIV compared to HIV-negative women and a reduction in loss to follow up (20% vs. 37%) when using a mobile phone tracking and recall system. [36]

**Table 4:** Model 2—Coordination between co-located clinics/specialists.

Study	Study design	Setting	Model of care	Integration	Screening n (% of those offered)	HIV positive	Treatment coverage	Selection bias
Odafe 2013	Cross-sectional	Secondary healthcare urban public hospital, *Nigeria*	All women attending ART were counselled on CaCx screening, those accepting were referred to the reproductive health unit for same-day VIA screening. Referred for colposcopy and treatment.	Coordination between ART unit and reproductive health unit with bi-directional referral and patient tracking system.	834 (96.5%)	100%	*NA*: *Screening only*	N/A
Horo 2012	Case -control with sub-cohort	Three ART clinics and a blood donor clinic, *Cote d’Ivoire*	Screening by mobile staff, referred for colposcopy if positive or inconclusive at ART clinic, follow-up and treatment at ART clinic.	Coordination between mobile staff and the ART clinic to provide screening and treatment for CaCx.	4,046	74%	414 referred for colposcopy, 36.5% (n = 151) did not attend. A systematic mobile phone tracking system reduced the loss to follow up from 36.5% to 19.8%.	N/A
Fink 2012	Cross-sectional	A hospital HIV clinic, *Argentina*	Screening: Pap smear and colposcopy	New weekly specific clinic for women living with HIV; care provided by HIV and gynecological specialists.	96	100%	*NA*: *Screening only*	N/A
Mwanahamuntu 2013	Cross-sectional	17 clinics and an outpatient surgery care center housing a Gynecologic Cancer Prevention Clinic, *Zambia*	‘See and Treat’: VIA and cryotherapy, refer cryotherapy-ineligible for evaluation and treatment to an outpatient surgery clinic located in a tertiary hospital.	Physical co-location of CaCx program clinics with HIV/AIDS clinics.	56,427	26.7%	*Not reported*	N/A
Ramogola-Masire 2012	Cross-sectional	Community and hospital-based HIV clinics, *Botswana*	“See and Treat”: VIA and EDI and cryotherapy. Cryotherapy ineligible referred for colposcopy/LEEP to local hospital. Complex lesions referred to specialized clinic, advanced cases referred to tertiary hospital.	Coordination between HIV clinic and CaCx screening community clinic in the same facility.	2,175	100%	253 received same-day cryotherapy. 575 were referred for further evaluation and treatment. 61.3% women received appropriate same-day screening and treatment without the need for recall or referral.	N/A
Parham 2010	Cohort	11 urban and four rural public health clinics, *Zambia*	“See and Treat”: VIA and cryotherapy, referred for histologic evaluation and clinical management. Follow-up visits for those undergoing cryotherapy or LEEP are encouraged at 6 weeks and 6 and 12 months.	Specialist nurses coordinate care independently in rooms co-located within 15 public health clinics.	21,010	31.3%	Of the women eligible for ablative treatment by cryotherapy, 78% (1603/2061) actually underwent treatment. A total of 75% (1095/1462) of HIV-infected women referred for evaluation complied. Less than 20% of women ever returned for their recommended follow-up visit.	High

Abbreviations: CaCx: cervical cancer, VIA: visual inspection with acetic acid, NA: not applicable, EDI: enhanced digital imaging, LEEP: loop electrosurgical excision procedure

Mwanahamuntu *et al*., presented the results from a cross-sectional study set in Zambia [39]. They evaluated the integration of new cervical cancer screening clinics into 17 public sector health clinics and a surgical center in Zambia, which delivered PEPFAR sponsored HIV care and treatment services at the same sites. HIV screening was also integrated into the cervical cancer clinics for women with unknown HIV status. These mutual linkages achieved greater efficiencies. Nurses were trained in cervical cancer screening and treatment of minor lesions. Digital images were used as an adjunct to screening and reviewed weekly with a gynecologist. For sustainability, peer educators were used for health promotion and as patient navigators to reduce loss to follow up, and the program was constantly refined through community feedback. Task-shifting helped overcome workforce shortages. The program screened 56,427 women (27% HIV+) during the study period (2006–2011). 28% of women were VIA+. Women living with HIV had 2.62 times higher odds of being VIA+ [AOR: 2.62 (95% CI: 2.49, 2.76, p<0.001] than HIV- women.

In another cross-sectional study, Odafe *et al*. evaluated a program integrating cervical cancer screening into existing reproductive and child health clinics co-located with HIV services in Nigeria [4]. Nurses and midwives in both units were trained in counselling, appointment setting and managing a bi-directional referral system. Women attending the HIV service were offered same day cervical cancer screening in the reproductive and child health clinic, and women with unknown HIV status referred for HIV testing. The program also offered screening for STDs. Electronic health record systems were created to support the referral program. The uptake of cervical cancer screening amongst women living with HIV (2009–2010) was 96.5% (n = 805/834). 7% (n = 52) were VIA+, 25% (n = 199) were diagnosed with an STD.

Three of the papers reporting this model described the use of digital images as adjunctive to the cervical cancer treatment, as a tool to improve training and quality [31, 38, 39]. The images were reviewed by gynecologists either remotely or in weekly sessions, together with the nurses. One of the studies, a cross-sectional study evaluating an integrated service in Botswana [31], found that 20% of women initially diagnosed with normal VIA results were recalled for assessment after the image review, finding 75.3% (95% CI, 72.5–77.8%) sensitivity of the nurse assessment and a 98.5% (95% CI, 97.6–99.1%) specificity.

### Model 3: Complex program of integration and coordination

Six studies, all set in Africa (Table 5), presented complex programs of integration of cervical cancer with HIV services with coordination across care pathways. A majority described integration of cervical cancer services into existing clinics providing HIV care. One study described the integration of HIV services into existing cervical cancer clinics [29]. All studies reported data on numbers of women screened for cervical cancer, three reported screening uptake as proportion of women offered cervical screening and five studies reported clinical screening results (Table 5). Three studies presented final cancer diagnoses. Only one study reported HIV-related measures, including uptake of HIV testing, HIV sero-positivity, and reasons for declining screening or not offering screening.

**Table 5:** Model 3—Complex program of integration and coordination described.

Study	Study type	Setting	Model of care	Integration	Screening n (% of those offered)	HIV positive	Treatment coverage	Selection bias
McCree-Hale 2011	Case series	Urban HIV clinics, *Tanzania*	HIV clinics provide Pap smear screening, slides sent to lab in external hospital, follow-up and treatment at national cancer center.	CaCx screening integrated into HIV a clinics, using existing staff. Coordination with external center and lay health workers to ensure follow-up of care.	1,440	100%	Of the 124 women with SIL, 5 (4%) presented for follow up and treatment at the national cancer center. The remaining 119 women had to be tracked using a district tracking mechanism comprised of trained lay health workers.	N/A
Khozaim 2014	Retrospective descriptive study	Four regional HIV clinics and child-maternal clinics, *Kenya*	‘See and Treat’: VIA and cryotherapy. Cryotherapy-ineligible evaluated by local gynecologists at mobile colposcopy service rotating once a month per site, biopsies evaluated at referral hospital and results reviewed by gynecologists at the clinics, referred for LEEP.	Integration of a public sector CaCx screening program into existing large HIV clinics. MoH working with NGO to train local staff and implement system of care across 4 regions. The collaboration provide logistic support, supply chain management, and screening rooms.	6,787	NA	206 women underwent cryotherapy, 754 colposcopy, 143 LEEP, and 27 hysterectomy. The overall loss to follow-up was 31.5%: 27.9% were lost after a positive VIA screen, 49.3% between biopsy and LEEP, and 59.6% between biopsy and hysterectomy/ chemotherapy.	N/A
Plotkin 2014	Cross-sectional	Government health facilities: The national consultant referral hospital, two regional hospitals, twelve district hospitals, and six health centers, *Tanzania*	**HIV:** HIV testing offered to all women unless they were HIV positive or (self) reported being tested in the last three months. If test positive, referred to the onsite HIV CTC. **CaCx:** “See and Treat”: VIA and cryotherapy, advanced cases referred. Three the facilities also offered LEEP for treatment of large lesions.	Integration of HIV testing within newly introduced CaCx screening and treatment services, located in the reproductive and child health (RCH) section of the facility. Coordinated referral between RCH and HIV CTC. Part of the Government National Strategy for CaCx prevention.	24,966 for CaCx; 11,819 (94%) for HIV	NA	*NA*: *Screening/testing only*	Low/unclear
Mungo 2013	Retrospective record review- before and after study	Family AIDS Care and Education Services HIV clinic, *Kenya*	VIA and colposcopy, LEEP treatment, advanced cases referred. Women treated with LEEP were re-screened with colposcopy at 6, 12, and 24 months, and those with CIN 2, CIN 2/3, or stage IA1 disease during follow-up were offered repeat LEEP.	Addition of LEEP treatment to CaCx screening services at a HIV clinic. Coordination with external pathologist to interpret biopsies, in-clinic gynecologist for clinical staging, and external hospitals for treatment.	4,308	100%	100% (39/39) stage IA1 patients and 95.1% (39/41) of all women with ICC accessed treatment.	High
Pfaendler 2009	Cross-sectional	13 primary care clinics and a tertiary care hospital, *Zambia*	‘See and Treat’: VIA and cryotherapy. Referral of patients for further evaluation: repeat VIA and punch biopsy or LEEP, with 6-week follow-up. Further referral as necessary.	Implementation of a referral and management system for cryotherapy-ineligible women needing further evaluation. HIV peer educators, HIV CTC staff, and nurses engaged in community awareness for CaCx screening among HIV-positive women.	8,823	41.5%	2,378 women were treated with cryotherapy and 1,477 were referred. 59.2% (875) of women referred kept their appointments. 748 women underwent LEEP.	N/A
Shiferaw 2016	Cohort	14 tertiary and secondary-level health facilities (5 allocated as Centers of Excellence), *Ethiopia*	‘See and Treat’: VIA and cryotherapy, ineligible referred for LEEP at Centers of Excellence. Counselled to return for follow-up.	Integration of CaCx screening and treatment into the HIV and AIDS care and treatment package, establish provider teams throughout five regions, build capacity, and promoted community education and awareness. Coordinated referral and patient follow-up between ‘see and treat’ sites and Centers of Excellence.	16,632 (99.4%)	100%	96.9% (1,481) of eligible women received cryotherapy on the same day as screening; 63.0% (80) of women referred for LEEP received treatment; 51.1% (614) of women expected to come for follow-up returned for screening 1 year later and were screened.	N/A

Abbreviations: CaCx: cervical cancer, SIL: squamous intraepithelial lesion, VIA: visual inspection with acetic acid, LEEP: loop electrosurgical excision procedure, MoH: Ministry of Health, NGO: non-governmental organization, CTC: care and treatment centers, CIN: cervical intraepithelial neoplasia, NA: not applicable

A cross-sectional study evaluating integration of HIV screening into 21 government health facilities providing cervical cancer services in Tanzania showed a good uptake of screening for both HIV and cervical cancer. [29] Nearly 25,000 women were screened for cervical cancer (2010–2013) and 60% of women with unknown HIV status offered HIV testing; 94% accepted and 5% tested positive. Limited access to HIV test kits was the main reason that not all women were offered HIV screening. The authors concluded that integrating HIV testing into cervical cancer screening facilities was acceptable to staff and patients and an effective way of reaching women with unknown HIV status. However, they also acknowledged that more support was needed to reduce logistical barriers, such as access to test kits and equipment.

A cohort study set in Ethiopia [32] analyzed the integration of VIA and cryotherapy in a single visit approach in 14 established secondary and tertiary health centers which offered HIV services. The program trained 77 health care workers (physicians, nurses, and midwives). To support the training, basic clinical and counselling guides, single visit standard operating procedures and quality management toolkits were provided. To ensure staff skills, bi-annual refresher training and quarterly visits were conducted using competence checklists. The program was feasible and acceptable to patients, as shown by high uptake of screening (99%) and 98% of eligible women received cryotherapy the same day. There was, however, high loss to follow up; the treatment rate for women referred for LEEP was only 63%. Moreover, only 51% (range: 30%-81% by regions) of VIA+ women returned for 1-year follow up. The proportion of eligible HIV positive women receiving treatment for major lesions varied by region from 4.8% to 86.7% suggesting inconsistent quality of service provision among providers or other variables which might have impeded access.

A retrospective descriptive study evaluated the integration of a public section cervical cancer screening program into HIV and family health clinics in Kenya. [40] This screened 6,787 women (2009–2011), finding 20% (n = 1331) VIA+ and 71% (n = 949) HIV+. 68 women had cancer, with an incidence of 414 per 100 000 women per year. The authors estimated that the screen-and-treat model, averted 349 cases from progressing to cancer. However, the loss to follow up was high (32%), and increased as treatment became more invasive. 28% were lost after a VIA+ screen, 49% between biopsy and LEEP and 60% between biopsy and hysterectomy/chemotherapy.

### Outcomes reported

Many studies described measures of clinical process and outcomes, with a majority presenting numbers of women screened for cervical cancer during the study period and some reporting uptake as proportion of women screened (Table 3). A majority also reported cervical cancer screening results. Only a limited number of studies followed up patients referred for treatment and reported final cancer outcomes. Most of these studies reported high loss to follow up for treatment, with some showing a reduction in loss to follow up through integration of treatment of larger lesions in a single visit, while others achieved improved results with mobile phone or other tracking systems. Other outcomes reported by studies, included improved access to gynecology examinations and STD screening. Process measures included staff satisfaction, patient acceptability and patient-perceived barriers. The reported facilitators and barriers to integration of cervical cancer and HIV care are summarized in Table 6. Four papers presented patient-perceived barriers to uptake of services including time, costs, fear of results and wanting to consult with their spouse/partner.

**Table 6:** Facilitators and barriers to integrated cervical cancer and HIV care. The tables shows barriers and facilitators mentioned in the results or discussion.

Facilitators	Barriers
Integration of the program within pre-existing healthcare infrastructures [26, 30, 34, 35, 39–41]	Lack of staff and skilled staff [5, 29, 30, 35, 40–42]
Task-shifting [34, 35, 39]	Lack of pathologists [39–41]
Evidence based cost-effective / low cost screening [30, 35, 38–40]	Staff fatigue [34, 35, 40]
Single visit approach (”see- and-treat”) [5, 26, 30, 32, 34, 35, 40]	High staff turnover, [29, 32, 35, 41]
Qualified staff, certified nurses with some medical training [38, 39]	Loss to follow up [24, 28, 29, 32, 35, 38–41]
Care coordinator [28]	Inconsistent supply of resources, incl. supplies and equipment [5, 29, 32, 34, 35, 39–41]
Staff willingness [30, 39]	Physical infrastructure [34, 35]
Training [26, 30, 32, 34, 35, 40]	Lack of medical records/electronic records [35]
Train the trainer models [22, 35, 39, 40]	Long waiting times for results, delays in access to treatment [24, 40]
Continuous education and/or supervision [22, 30, 32, 34, 35, 39, 40]	Limited treatment capacity [5]
Screening algorithms and/or protocols [30, 32, 40, 42]	Lack of recall/follow up systems [5, 41]
Digital camera for training and quality improvement [31, 38, 39]	Lack of financial incentives to providers [29]
Capacity building for health care workers [4]	Inconsistent quality between providers [32]
Developing referral system [5, 42]	Failure to screen for HIV /missed opportunity [35]
Bi-directional referral: HIV and reproductive health services [4]	**Patient barriers:**
Electronic medical records system [4, 30]	Treatment and transport cost [32, 36, 42]
Phone-based tracking/call and recall system [28, 36, 40]	Long time to wait for treatment[42]
Renovation of facilities, appropriate screening rooms [35, 38, 40]	Long transport [5, 32]
Stakeholder engagement, community participation [4, 32, 39]	Lack of time [36]
Health promotion targeting patients [30, 32, 39, 41]	Fear of cervical cancer diagnosis and treatment [36]
Peer educators [30, 39, 41]	Fear of HIV test results [29]
Low transport costs [41]	

### Risk of bias

We screened all studies and found only five eligible for risk of bias assessment as they presented evaluative data [29, 30, 34, 38, 42]. Mungo *et al*. present a before and after study with high risk of bias due to selection (no randomization, no concealment, selection of groups not done at the same time), attrition (incomplete follow-up and outcome data), and information bias (record review). Parham *et al*. also had high risk of bias. This cohort study had high loss to follow-up and low confidence in the validity of the assumptions used for predictive values, progression rates, and cure rates in the model (these were based on few and highly heterogeneous publications). Although the selection bias in Plotkin *et al*. appeared to be low, with all records at the facilities being reviewed, adequate information was not provided in this cross-sectional study to assess other forms of bias. Huchko *et al*. describe a cross-sectional study and Martin *et al* a retrospective record review. Neither study stated the selection criteria for women to be offered screening and Huchko *et al*. collected data from health records of unclear quality.

### Measures of effectiveness of integration

Five studies formally evaluated the integration of HIV and cervical cancer services (Table 7). These included two cross-sectional studies [29, 30], two retrospective record reviews, one of which was a retrospective record review before and after study, and a cohort study.

**Table 7:** Results of the studies evaluating integration.

Study	Objective	Setting and sample size	Study design	Clinical & behavioral outcomes	Process outcomes	Risk of bias
Huchko 2011	To evaluate outcomes of cervical cancer screening within HIV care and treatment	District hospital and HIV clinics in western Kenya. n = 4,186 HIV+ women attending the clinics. n = 23 clinicians interviewed about the program	Cross-sectional	• Acceptability of screening: 87% (3642) of those offered accepted; 96% of whom accepted screening during the current visit. • Accessing new population: <1% (18) women reported having had screening in the past.	• Reasons for declining screening included “needing to talk with their husband”, “being on their menses”, “needing to think about it”, and expressing fear of the speculum exam. • Staff reported a high level of satisfaction with their training and their role in implementing CaCx screening in the clinic. Main challenges reported were related to infrastructure limitations and perceived patient barriers.	• Overall: unclear *selection*: unclear • *diff misclas*: NA • *non-diff misclas*: unclear • *confounding*: NA
Martin 2014	To evaluate a cervical cancer prevention project in Guyana and to identify lessons learned to inform the improvement of cervical cancer prevention programs.	Cervical cancer prevention sites including HIV care and treatment sites and a hospital across 9 regions in Guyana. n = 21,597 women (HIV+ and HIV-) attending the sites.	RRR	• Wide coverage of screening: 95% (21,597) of HIV+ women enrolled in care and 17% of women aged 25–49 years in Guyana were screened. • A smaller proportion of HIV+ women received immediate cryotherapy compared to HIV−/unknown women (73% vs 86%; P < 0.001). • Of those who postponed treatment, similar proportions returned for cryotherapy (HIV+, 60%; HIV−/unknown, 62%; P = 0.73)	• At study period end, 49 (69%) trained providers were still offering VIA, cryotherapy and/or LEEP services. • Non-physician providers were more likely to continue providing services than physicians (80.5% vs 53.3%). • Most programmatic challenges were related to systemic rather than technical/ clinical issues.	• Overall: Unclear, *selection*: unclear • *diff misclas*: NA • *non-diff misclas*: unclear • *confounding*: NA
Mungo 2013	To evaluate the effect on treatment follow-up of offering LEEP in-clinic compare with by referral.	HIV clinic in western Kenya. N = 4,308 HIV+ women	RRR: Before and after study	• Increased access to treatment after addition of immediate LEEP in-clinic services *[100% (39/39) stage IA1 patients and 95*.*1% (39/41) of all women with ICC accessed treatment]* compared with referral to a local hospital *[26*.*7% (4/15) stage IA1 patients and 35*.*5% (6/17) of all women with ICC accessed treatment]*.	• When offered LEEP in-clinic or referral for the treatment of stage IA1 disease, all eligible women chose LEEP performed in-clinic at no cost.	• Overall: high^1^ • *selection*: high *performance*: NA detection: unclear/high, attrition: high, reporting: unclear
Parham 2010	To analyse clinical outcomes and modelled program effectiveness among HIV-infected women by estimating the total number of CaCx deaths prevented through screening and treatment.	11 urban and 4 rural public health clinics in Zambia. n = 21,010 women (HIV+ and HIV-) attending clinics	Cohort	• One cervical cancer death prevented per 46 (corresponding range: 28–68) HIV-infected women screened by the program (142 prevented deaths among 6572 screened).		• Overall: high • *selection*: high • *diff misclas*:NA • *non-diff misclas*: low • *confounding*: NA
Plotkin 2014	To provide a rough measure of the success of integration of HIV testing into cervical cancer screening, in order to inform scale-up of cervical cancer screening services in Tanzania.	21 health facilities across 4 regions in Tanzania. n = 24,966 for CaCx screening and 11,819 for HIV screening	Cross-sectional	• Acceptability of testing: 94% (11,819) of those offered accepted HIV testing • 5% (582) of those tested learned for the first time that they were HIV-positive.	• The proportion of clients offered HIV testing started out high (averaging 86% in 2011), and fell to 62% in 2012, and 55% in 2013. • Reasons for declining HIV test: most (96%) said they ‘did not feel ready’ or ‘were afraid’, 3% said they wanted to consult their husband, while the remaining 1% did not specify a reason. • Unavailability of HIV test kits at the facility was the most common reason for a CaCx screening client not to be offered an HIV test (71% of 6,321 cases).	• Overall: unclear/low • *selection*: low *diff misclas*: NA • *non-diff misclas*: unclear • *confounding*: NA

Abbreviations: NA: not applicable, RRR: retrospective record review, CaCx: cervical cancer, diff misclas: differential misclassification, non-diff misclas: non-differential misclassification

^1^ Risk of bias assessed as a randomized control trial for whether there is a difference between referral-only (before) and in-clinic treatment (after)

The before and after assessment involved the transformation of a system of cervical cancer screening in a HIV clinic with referral for treatments to one that included on-site Loop Electrosurgical Excision Procedure (LEEP) treatment within the HIV clinic and referral only of complicated cases [42]. Addition of immediate LEEP in-clinic services increased access to treatment (from 26.7% to 100% for women with stage IA1 disease (<3mm deep and 7mm wide, with no spread) and from 35.5% to 95.1% for all women with invasive cervical cancer) compared with when patients were instead referred to a local hospital. Patient preference for the integrated in-clinic service was also described, with women living with HIV choosing to receive LEEP treatment in the HIV clinic instead of the local hospital.

Parham *et al*. present a cohort study that modelled the effectiveness of their cervical cancer prevention program in Zambia, which introduced cervical cancer screening and treatment for women living with HIV, by estimating the total number of cervical cancer deaths prevented [38]. They estimated that the program prevented one cervical cancer death per 46 HIV positive women screened. It is not reported, however, whether the introduction of cervical cancer screening and treatment, independent of integration with HIV services, would have a similar effect on preventing death.

A cross-sectional study looked at the proportion of cervical cancer screening clients who were offered HIV screening through an integrated care program over time to evaluate the sustainability of the integrated service [29]. Although the acceptability of HIV screening among cervical cancer screening clients was high (94%), the proportion of clients offered HIV testing dropped over time (from 86% in 2011 to 55% in 2013), suggesting a lack of sustainability of the integrated screening program which could have been due to a variety of factors.

Two studies, a cross-sectional one [30] and a retrospective record review [34], assessed the process of integrating services [30, 34]. Huchko *et al*. used a questionnaire to ask staff about the implementation of VIA screening and LEEP treatment services at Family AIDS Care and Education Services clinics [30]. Staff reported a high level of satisfaction with their training and their role in implementing cervical cancer services in the HIV clinic. The main challenges reported were related to infrastructure limitations and perceived patient barriers (Table 6). Martin *et al*. conducted an evaluation of the implementation process to assess the sustainability of their integrated ‘screen and treat’ cervical cancer program through provider and stakeholder interview, chart review, and service statistics [34]. They concluded that the single-visit cervical cancer screen-and-treat program was feasible, effective and sustainable. This was based on findings that, at the end of the study period (January 2009 to June 2012), trained providers were still offering VIA, cryotherapy and/or LEEP services. Moreover, 95% of women living with HIV women enrolled in care at the clinics were screened for cervical cancer and, although a somewhat lower proportion of HIV-positive compared with HIV-negative women received treatment (73% immediate and 60% postponed versus 86% immediate and 62% postponed), overall treatment coverage was high. The study also found that non-physician providers were more likely to continue providing services than physicians (80.5% versus 53.3%).

### Implementation of integrated care programs

In most programs studied, nurses and/or midwives were the main healthcare workers responsible for cervical cancer screening and care coordination, with other cadre also engaged for specialist functions such as supervision, pathology, and advanced care [4, 5, 22, 26, 29, 31, 35–38, 40, 41]. Some studies describe the involvement of a wide range of professions and cadre to ensure integration of services and follow-up of patients. For example, in western Kenya, Khozaim *et al*. describe local nurses, gynecologists, oncology-gynecologists, laboratory staff, pathologists, and lay people all being involved in the delivery of integrated care. Additionally, specialists from the USA were involved in continuous supervision of healthcare staff. Martin *et al*. describe services in Guyana being shifted to non-physicians for scale-up of high-quality cervical cancer prevention program nationally. Some studies recruited lay people for community engagement activities, peer education, and to help with patient follow-up [24, 37, 40, 41].

Training was highlighted as a key feature in setting up programs of integration and expansion of health services. For the most part, training was delivered by program partners as a mixture of didactic classes and practical sessions [4, 5, 30–32, 35, 36, 41]. Some studies also describe a process of on-site continual assessment and supervision [5, 32, 40]. Other programs took a training of trainers approach; in Zambia, Mwanahamuntu *et al*. describe a train-the trainer model where nurses served as educators for their peers. In Kenya, Khozaim *et al*. describe key nurses undergoing training in cryotherapy and cervicography. These nurses then trained and mentored other local Ministry of Health nurses who staffed the cervical cancer screening services in their clinics. Only two studies found that programs used international guidelines to support training and clinical practice. Odafe *et al*. used the practical manual on visual screening for cervical neoplasia published by World Health Organization (WHO) -International Agency for Research on Cancer (IARC) and Khozaim et al describe using WHO guidelines on referrals and treatments. Other studies describe purposefully developing guidelines, protocols and clinical algorithms as part of the program [26, 30, 34].

In addition to the training of health staff and need for clinical guidelines, other resources were identified as necessary for implementation of integrated services, including infrastructure, equipment, and additional personnel along the care continuum, including sufficient pathology support for timely access to diagnostics. Resources described include: material to provide education and support to patients [5, 28, 34, 40]; staff to operate the referral system [28, 30]; staff to coordinate integration[28]; forms or electronic medical records [4, 36]; and medical and adequate infrastructure, including consistent electricity and water supply.

All studies utilized existing infrastructure as a platform for integrating services. Some used infrastructure that had been previously established through existing technical assistance for HIV programs [26, 30, 31, 35, 36, 40]. For example, Moon *et al*. describe a program in which Friends in Global Health (FGH) utilized infrastructure that had been established through the roll-out of Zambia’s PEPFAR-funded HIV care and treatment program. FGH supported the introduction of cervical cancer screening by providing logistical and training support for screening, including equipment purchase and distribution, minor facility renovations, in-service training of national health system nurses and doctors, weekly clinical mentoring and on-the-job training of nurses tasked to perform VIA and cryotherapy, and assistance with data collection and analysis.

## Discussion

In a systematic review of the literature to evaluate attempts to integrate HIV and cervical cancer services, we identified three models of integration: within clinic integration using existing staff; coordination between co-located clinics/specialist; and complex programs of integration involving coordination of many specialists/clinics. Overall, evidence suggests that the provision of integrated services was feasible, safe, and acceptable to both staff and women attending the health facilities. Uptake of cervical cancer and HIV screening was high in all models described, but loss to follow-up for cervical cancer treatment was a challenge in most studies.

Most studies described integration of cervical cancer screening using visual inspection methods into existing HIV clinics, through training of existing staff in visual inspection techniques, and treatment of minor to larger lesions. This model is potentially the most cost-effective, as it reduces the need for additional specialist staff and clinics and enables integration of both HIV and cervical cancer services in a single visit appointment. There is, however, a lack of data available on the cost-effectiveness of these integration approaches.

Integration of screening and treatment into a single visit reduced loss to follow up. However, limited evidence is available on the best approach to providing these additional services or on the long-term impacts of integration of services on patient outcomes and health system strengthening. There was a limited number of studies that formally evaluated the integrated services and most studies were set in Africa describing the introduction of new cervical cancer services. Thus, a comprehensive evaluation and comparison of models of integration was not possible. It was, however, noted that there were no notable differences in facilitators and barriers between the models.

### Barriers and facilitators to integration

All but one study described the introduction of new services as part of the approach to providing integrated care. In doing so, many barriers and facilitators to the introduction and integration of services are described (Table 6). That the reported facilitators and barriers were similar in all models may be partly attributed to the fact that most studies were in Africa, in settings with no or limited access to cervical cancer screening prior to integration of the service. For all models, there was a need to train staff in new screening techniques, even in model two, which described co-location of cervical cancer and HIV services. Many of the challenges documented were not necessarily specifically related to the integration of services, but general health systems challenges experienced in many low-resourced settings [43].

Several distinctive facilitators where identified. The use of pre-existing infrastructure, such as government-funded clinics, staff and services already targeting women living with HIV, [5, 26, 30, 34, 35, 37, 38, 40, 41], together with the use of HIV funded services, [35, 38] were key facilitators to integration together with the single visit approach. Training of staff was another key enabler for integrating the new services in all models, together with access to continuous training and supervision for quality and sustainability [30, 32, 34, 35, 39–41]. Access to screen and treat algorithms and guidelines were useful tools to support the training of health workers, [30, 32, 40]. Access to electronic patient records [30, 38] facilitated quality by enabling tracking of patients and results through the care pathways. Stakeholder engagement, not only with staff but also with patients and communities, through talks, and community health promotion campaigns, were other important aspects to sustain uptake [30, 38]. Peer-education, together with counselling, were other important facilitators.

There were many barriers identified across the models, predominantly from the studies set in low-resource settings, including logistical and chain management support [28, 29, 31, 32, 37, 40, 41] and access to consistent water, electricity, and equipment [5, 30, 35]. Despite studies reporting staff satisfaction with the integrated services, increased staff workload [34, 35], shortage of staff [5, 30, 35], and high staff turnover [29, 32, 34, 35, 40, 41] were other key barriers reported, for both within-clinic and co-located services. There were few studies reviewing sustainability over time and in different settings, with some indicating variation over time among different provides. Pfaendler *et al*., found that only a quarter of the staff initially trained in the single visit approach were still working at the sites when assessed and four sites lacked a specialist gynecologist. Other barriers frequently reported highlighted the need for strengthening of health systems across the care pathway when setting up a new service, including ensuring sufficient laboratory capacity [29, 31, 34, 35, 40, 41] for timely results and effective patient record systems [5, 30, 35]. Support for the overall clinic infrastructure and human resources should, thus, be prioritized to improve the sustainability of integrated care programs.

Several studies also reported high loss to follow-up [37, 38]. Reasons reported for loss to follow-up were similar across the studies; cost of treatment [36, 42] or transport [5, 29] together with long journeys, long waiting times [42] and fear of HIV and cervical cancer diagnosis [29, 36] were the main patient related barriers reported. One study reported the need to consult with the woman’s partner as another barrier. However, some studies found ways to reduce loss to follow-up through mobile phone tracking systems [36] and appointment reminders [40] or active follow-up by trained lay health workers [24, 40]. There is a need for a better understanding of the extent to which women, facing many competing pressures, including day to day survival in many settings, must make trade-offs and, in this process, what value do they place on an intervention where the cost is immediate but the benefits are a long time in the future. It is well recognized that the characteristics of individuals can impact substantially on their time preferences[44].

### Study strengths and limitations

One of the strengths of our review was the inclusion of studies from a varied range of databases and conference archives, which served to increase the number of papers from low- and middle-income countries. A limitation of our review is that most papers were descriptive, which although providing insightful knowledge on strategies and approaches, constrained us from assessing the effectiveness of programs. It is likely that other attempts have been made to integrate HIV and cervical cancer services but have not been published. Most studies found were from Africa, with limited studies from other geographical areas with different health systems. Additionally, there may exist publication bias if studies with null or negative findings were less likely to be published.

### Implications for research

The evidence currently available on the integration of HIV and cervical cancer services, mostly describes the introduction of new cervical cancer services into the HIV services setting rather than evaluating integrated versus non-integrated services. Only five of the 23 studies reviewed evaluated the effectiveness or sustainability of the integrated services. One of these five studies compares ‘more’ with ‘less’ integration of services; reporting positive findings on acceptability and effectiveness of the more integrated model [42]. The remaining four studies examine the process of integration and the sustainability of the model of service delivery. Furthermore, the outcomes explored across studies were mostly immediate clinical and process outcomes (Table 3).

There is a need for high quality and robustly designed studies that seek to evaluate and compare integration models (including comparison with non-integrated services) in terms of their long-term impact on patient outcomes, including HIV disease progression and treatment adherence, as well as cervical cancer morbidity and long-term outcomes, but also system-level parameters, such as cost-effectiveness and sustainability. There is a need for evaluation to be incorporated within the design and implementation of innovative programs that can establish causality, including the different elements of the new program.

### Implications for policy and service delivery

Despite the limited evidence available, descriptive studies on the introduction of cervical cancer services with HIV services have provided helpful insights into what has worked and what challenges remain in expanding services for all women, but especially women living with HIV. These findings highlight the need to incorporate strategies to reduce loss to follow-up. Ensuring adequate infrastructure, human resource strengthening, and logistical support across care pathways is important to promote uptake of services. Many of the challenges described in the studies are common in low-resource settings. When exploring the feasibility of integration of health services, it is important to acknowledge that these services are embedded within a system of care. Restructuring of health services should be considered as a complex intervention, and should be researched and evaluated, including through improved routine monitoring and using multidisciplinary and comprehensive approaches to understanding the wider-ranging effects of differing approaches to providing health services. Furthermore, this comprehensive approach should be extended beyond health services, through education at healthcare and community level, including comprehensive sexual education, information campaigns about the benefits of prevention, and addressing concerns about vaccination and screening services. Moreover, involvement of community health workers as well as healthcare providers are important to promote prevention and uptake of screening and treatment services for both cervical cancer and HIV.

### Conclusions

Several approaches to the integration of HIV and cervical cancer services have been described, ranging from the training of existing staff in cervical cancer screening and treatment to complex coordination of care between HIV and cervical cancer specialists and clinics to ensure appropriate screening, treatment, and follow-up of patients. Integration programs are mostly reported for low- and middle-income settings in sub-Saharan Africa, where HIV prevalence is high and the provision of cervical cancer services remains low. Most studies were conducted in partnership with national governments and led by NGOs.

Integration of HIV and cervical cancer screening and treatment is feasible and acceptable to staff and patients and has the potential to improve uptake of screening for women living with HIV. However, further research is needed to evaluate the impact of these models on treatment adherence and long-term outcomes. The results also highlight a need to strengthen health systems along care pathways, with an emphasis on staffing, training, and adequate supply of equipment.

The links between cervical cancer and HIV are a reminder that the needs of people living with HIV are complex and often demand integrated solutions. Those settings with the highest burden of HIV are often those with less integrated care services and health systems structured around provision of care for acute conditions. The growing burden of chronic conditions, both infectious and non-infectious, in resource-limited settings, will require further research into how to most effectively and efficiently restructure health services to improve access and quality of care.

## Supporting information

S1 Table PRISMA checklist. (DOC)

S1 Fig Study flow diagram. (TIFF)

S2 Fig Map of countries represented by cervical cancer services offered. (TIFF)

S3 Fig Model diagram for the integration of HIV and cervical cancer services. (TIFF)
