# Supplementary figures and images for: Integrating cervical cancer with HIV healthcare services: A systematic review
Source: PLoS One. 2017 Jul 21;12(7):e0181156. doi: 10.1371/journal.pone.0181156 (PMC5521786; doi:10.1371/journal.pone.0181156)

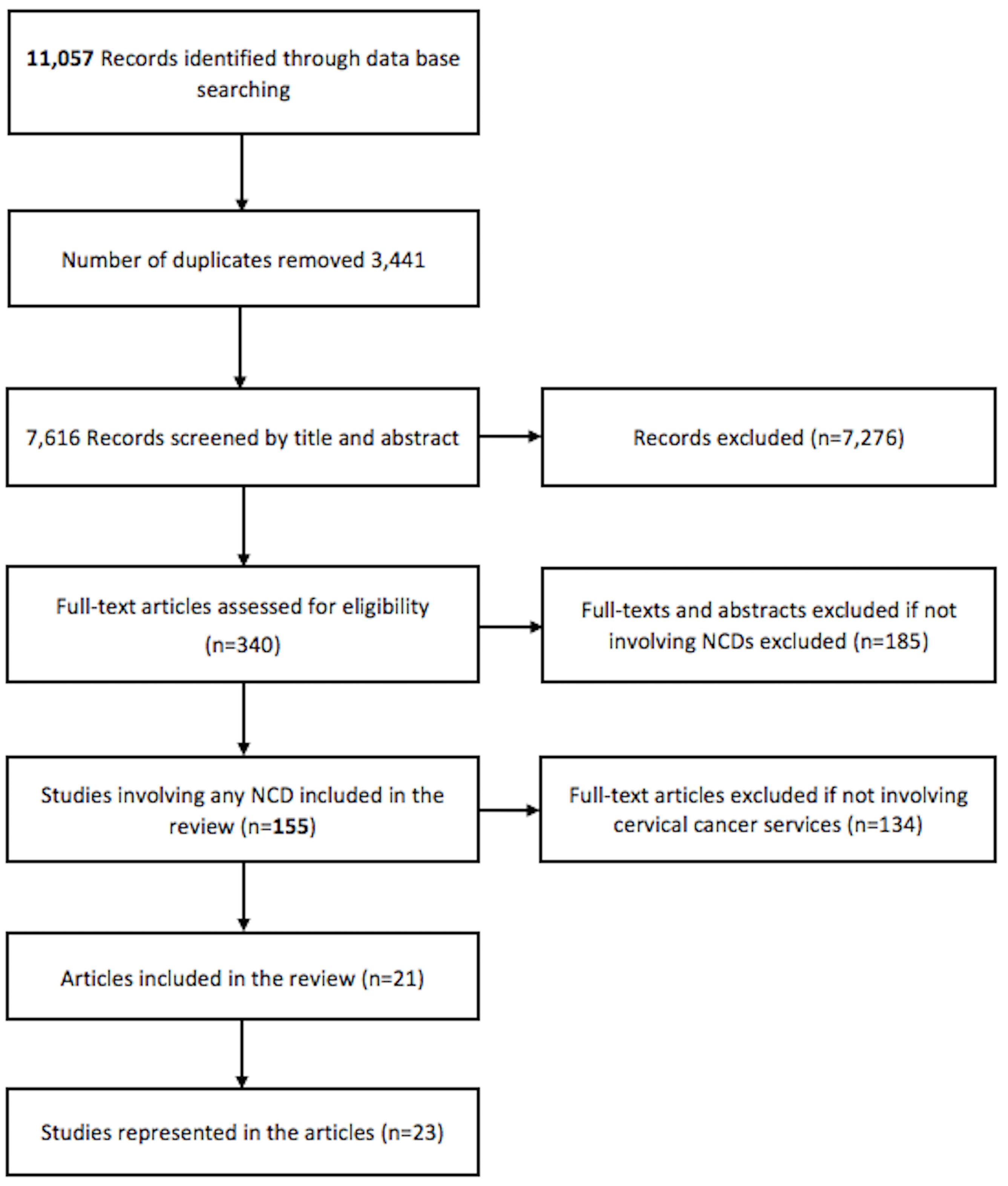

Supplement: S1 Fig — (TIFF) [file pone.0181156.s002.tiff]

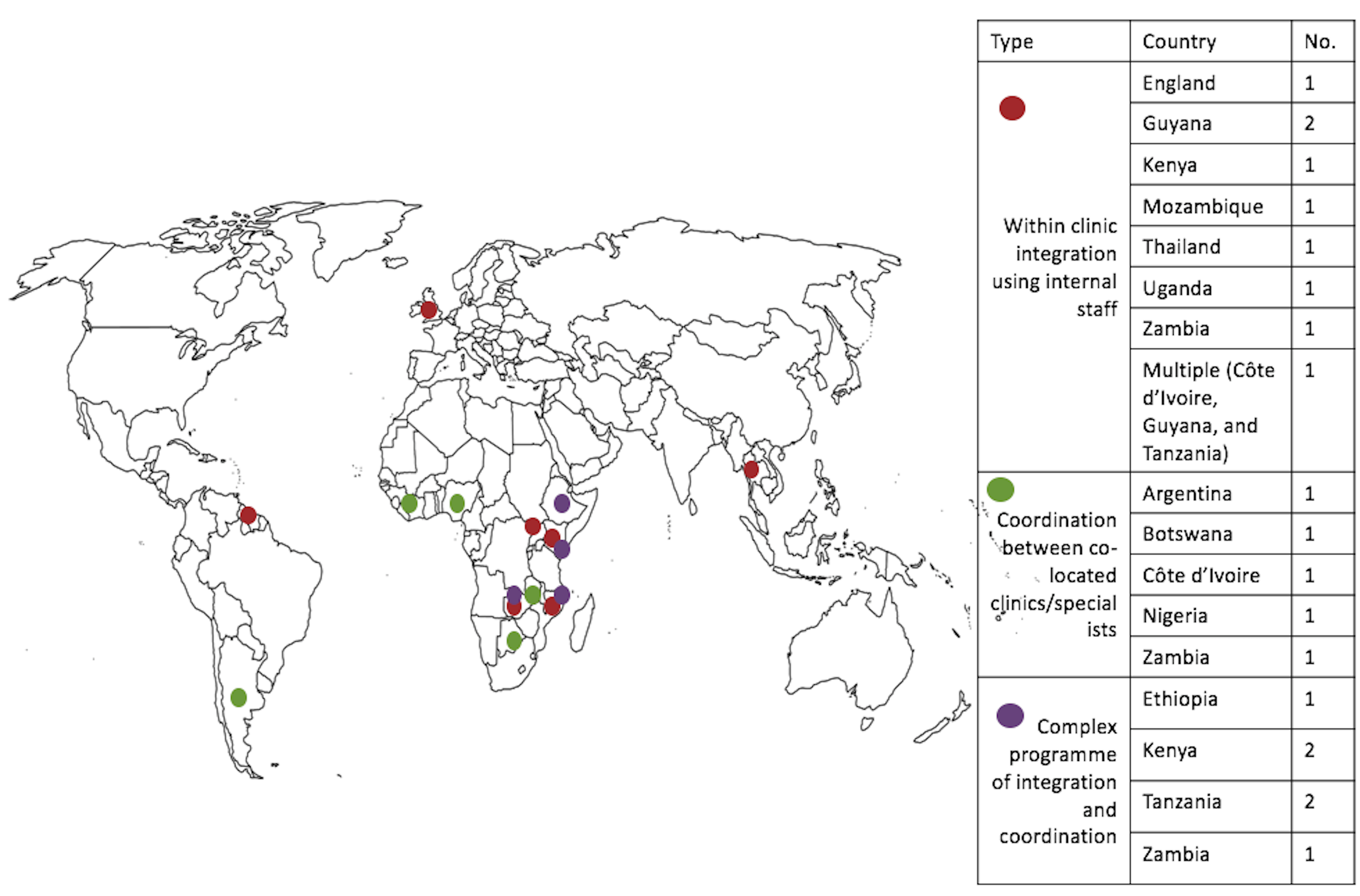

Supplement: S2 Fig — (TIFF) [file pone.0181156.s003.tiff]

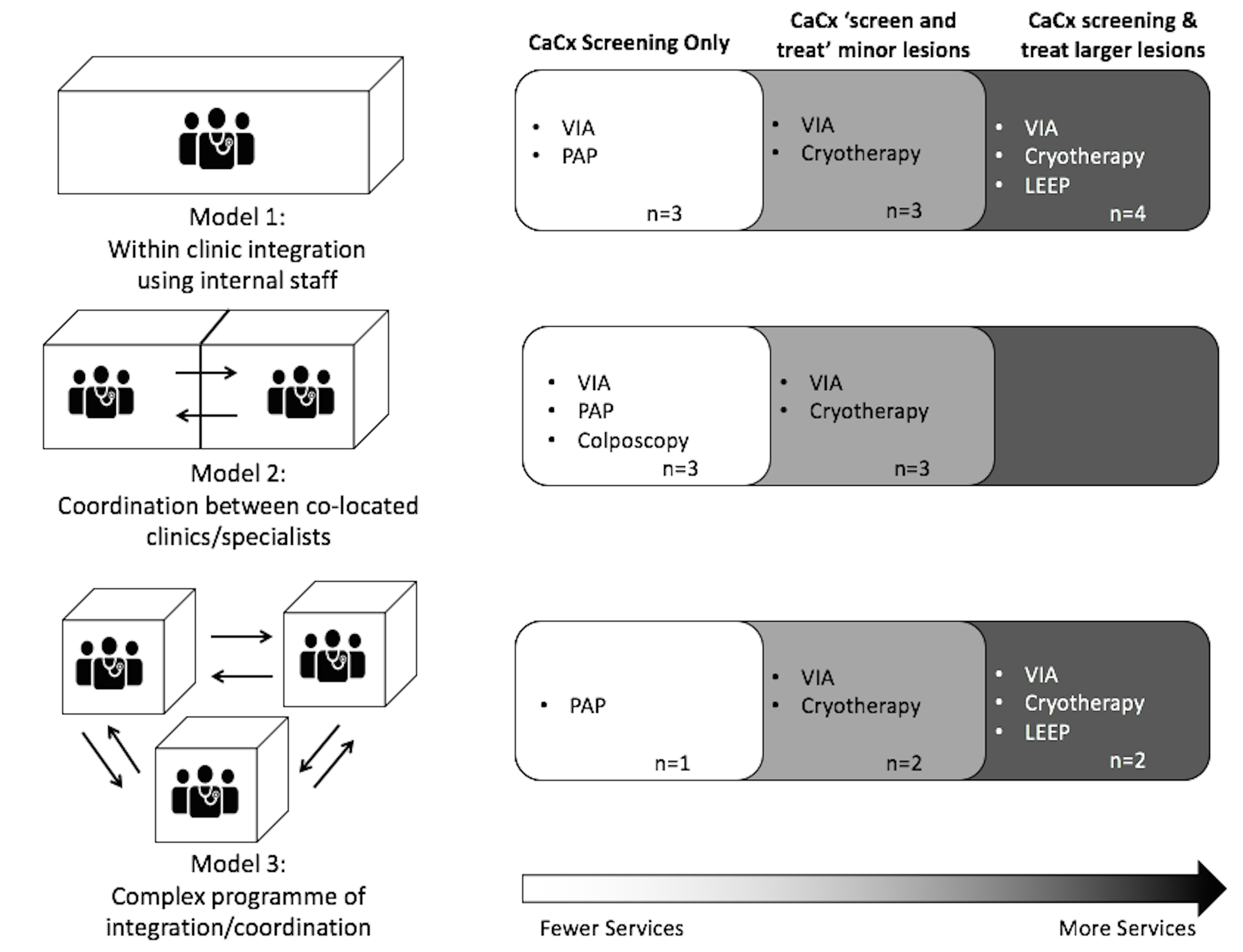

Supplement: S3 Fig — (TIFF) [file pone.0181156.s004.tiff]
